# Death anxiety as mediator of relationship between renunciation of desire and mental health as predicted by Nonself Theory

**DOI:** 10.1038/s41598-022-14527-w

**Published:** 2022-06-17

**Authors:** Yi-Fen Kuo, Yun-Ming Chang, Mei-Fang Lin, Ming-Lung Wu, Yung-Jong Shiah

**Affiliations:** 1 Graduate Institute of Counseling Psychology and Rehabilitation Counseling, National Kaohsiung Normal University, No. 116, Heping 1st Road, Kaohsiung, 802 Taiwan; 2 Department of Psychology, School of Educational Science, Minnan Normal University, Zhangzhou, China; 3 Fujian Key Laboratory of Applied Cognition and Personality, Zhangzhou, China; 4 Teacher Education and Careers Service, National Kaohsiung Normal University, Kaohsiung, Taiwan

**Keywords:** Psychology, Health care

## Abstract

In the present paper, we report two studies examining the relationships among renunciation of desires, death anxiety, and mental health. In the first study, we constructed the Desire Questionnaire (DQ), which measures success in renouncing certain desire. The DQ has satisfactory psychometric properties. In the second study, 501 adults from a Chinese society ranging in age from 17 to 84 years (*M* = 35.58, *SD* = 14.76) completed the DQ, the Death Anxiety Scale (DAS), and the Chinese Health Questionnaire (CHQ), which measures mental health and the presence of psychiatric symptoms. As predicted, DQ had significant negative correlations with both DAS (*p* < 0.05) and CHQ (*p* < 0.01). DAS had a significant positive correlation with CHQ (*p* < 0.01). In the linear mediation model, DAS was found to partially mediate the relationship between DQ and CHQ (*β* = − 0.18, *p* < 0.001). These results suggest that the negative effect of poor control of desires on mental health can be partially accounted for by death anxiety. These findings imply that training in eliminating desires can be a successful strategy to improve mental health. These results also support the Nonself Theory as a theory of death anxiety and show its relevance to the relationship between nonself and mental health.

## Introduction

The role that drawing upon Buddhist wisdom can play in improving mental health has emerged as a topic of psychological research and clinical application during the past 20 years^1–5^. Based on the Nonself Theory^6^, the core concept in Buddhism is nonself, a state through which one can achieve the realization that self is impermanent and that its nonappearance is intrinsic to human existence. The state of nonself is detachment from the world and from self^5,7,8^, particularly from egoism and desires*,* while one’s self-concept, or *atama-graha*, is maintained^6,7^. If an individual considers self, as well as objects and events, as independent and permanent, he or she can develop a wide range of psychological problems^7,9^. Self-cultivation is a process that wipes away the illusion of self^7,10^, involving practicing meditation, renouncing desire, exhibiting compassion, and pursuing realizing Buddhist wisdom^6^.

Previous studies have focused mainly on the effects of meditation and compassionate behaviour on mental health. For example, meditation has been found to be associated with several important mental health indicators, such as emotional stability^11^, positive emotion^12,13^, attention^14,15^, and psychological well-being^1,5^. There also has been research confirming a relationship between compassion and mental health, and self-compassion has been shown to be a strong predictor of symptom severity, including that of depression and anxiety^16^. It also has been shown to help one cope with stress and demanding life events^17^ and better adjust to the emotional impact of illness^18^. However, to the best of our knowledge, the possible effect of renunciation of desire on mental health has not been investigated. As mentioned above, renunciation of desire is a function of nonself. Thus, it is logical to propose that it has a positive relationship with mental health. Note that there are five well-known precepts in Buddhism: no killing, no harmful speech, no sexual misconduct, no intoxicants, and no stealing. These five precepts refer to ways persons towards the nonself state renounce desires by performing negative and positive duties^6^. This is why these precepts were chosen as subcategories for the development of Desire Questionnaire (DQ) in the present paper.

## The association between nonself, desire, and death anxiety: from self to nonself

There is no doubt that death is the paramount threat to the self or to one’s identity. Our awareness that we will eventually die is a tacit recognition that life is fragile and that the self will someday disappear. The death anxiety causes our terror that affects everyone by pushing one to seek meaning in death^19–21^. The aim of this quest for meaning is to overcome death anxiety. It is important to understand that this meaning creation provides us with information about the presence of stable patterns and coherence of expectations and behaviors in the environment^22^, and it helps us cope with life’s adversities^23–25^.

In our opinion the most well-reasoned theory attempting to explain what death means to people is the Terror Management Theory (TMT)^19,26,27^. The core postulate in the theory is that we draw upon our self-esteem, a feeling of self-importance or significance in the world, to overcome the fear of death. Self-esteem in TMT is a culturally grounded concept in the sense that we enhance it by convincing ourselves that our behavior meets the norms dictated by our culture^28^ and that we are making important contributions to this culture, that the life we live is valuable and meaningful. TMT maintains that what drives human beings more than anything else is our need to conquer death anxiety. It is the role of self-esteem to activate our psychological defense mechanisms against death^29^.

For more than 2500 years Buddhists have practiced a distinctive approach to coping with death anxiety, with the ultimate aim of overcoming and eliminating it^6,7,30^. It is of academic interest to explore the possibility that Buddhism provides an alternative perspective on the meaning of death and how to manage death anxiety. Thus, a major aim of this paper is to present a Buddhist perspective on the meaning of death. Buddhism shares with TMT the proposition that the awareness of death leads to anxiety. But Buddhism holds a different view than TMT on self-esteem and its role in dealing with death anxiety^30,31^. In contrast to self-esteem, nonself is a state in which a person has a sense of egolessness, which reflects awareness and realization of nonself^32,33^. The core premise of Buddhism is that ordinary (delusional) self, as well as self-esteem, are impermanent and dependent^6^. Self arises from brain processes of a psychological nature, such as perception, cognition, and motivation^6,34,35^. These psychological processes should not be confused with self per se, but they are what persuades us that self really exists, which in turn requires us to assume the reality of stimuli from a real external world, which is what the psychological processes respond to. It’s analogous to how we might think of a car. The myriad parts of the car, such as the engine and the transmission, are not the same as the car, but they are necessary for the car to function and the car would not be a car without them. In other words, everything is a collection of other things. Likewise, memories of some of our conscious experiences come together to mould our self-identity. But if self-identity exists, there must be a self for the person to identify with; in other words, self exists. But none of these constructs, and that includes self as well as self-identity, self-esteem, and self-whatever, has an intrinsic nature or substance. As Dalai Lama^7,36^ put it, self in reality is not as we perceive it to be; the delusional conviction that our perception of self is accurate is a product of our mental impressions, which also are not real. Buddhists have long believed that nonself conquers death anxiety^31,37^.

The threat of death is always present and occurs in a variety of forms. If we succeed in maintaining or boosting self-esteem for the purpose of dealing with death anxiety, a new threat may supplant the previous one. We need countless action to boost and keep self so it can cope with death anxiety. TMT states that the prospect of death threatens the self-esteem. It is logic to infer if there is no self or identity, death can’t threaten the nonself. Thus, there is no death anxiety. For this reason, based on the Nonself Theory^6^, the state of nonself helps one cope with death anxiety. Patients in hospice care who volunteered to practice Buddhist wisdom were found to have a decreased fear of death^38^. The same was found for a bone cancer patient^39^.

Accordingly, it is logical to assume that renunciation of desires approaching the nonself state is associated with a reduction in death anxiety. This was the second hypothesis tested in the present research.

## The role of death anxiety between desire and mental health

Death anxiety has been found to have a significant negative effect on mental health^40^. Death anxiety was also found to negatively influence the assessment of one’s mental health in a sample of deployed soldiers^41^ and to be a strong predictor of mental disorders^42^. Death anxiety has been shown to have significant positive correlations with depression and anxiety symptoms^43^ and post-traumatic stress disorder^44^. Taken together, these studies suggest that both desire and death anxiety are associated with mental health and that death anxiety can be overcome by minimizing self to attain the state of nonself. Renunciation of personal desires, for example, is one of the best strategies to deal with inevitable death anxiety.

In Buddhism, self is considered to be the primary cause of death anxiety^7,30,31^. Recall that according to TMT, the death cannot threaten self-esteem if there is no self or identity, because there is no self-esteem that death can coerce. Based on the Nonself Theory^6^, there would be no unhappiness and no anxiety, but rather the greatest equanimity, contentment, happiness, and with no room to suffer the pain caused by death and life’s adversities.

**Figure 1:**
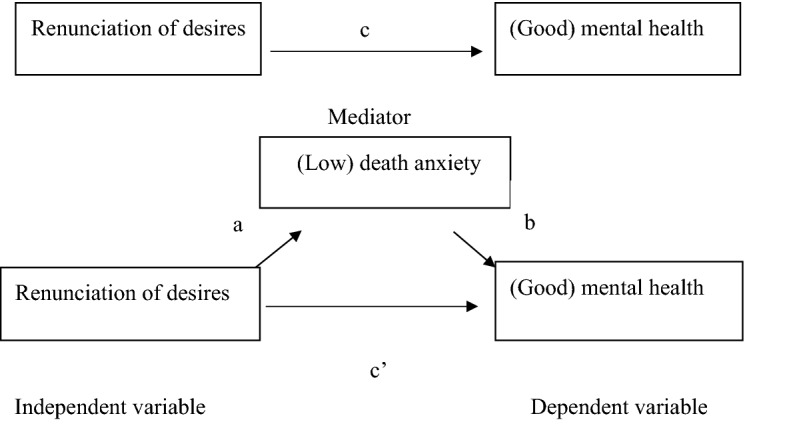
Mediation model for Study 2.

Accordingly, based on the assumption that death anxiety develops as the renunciation of desires is channelled by cultural, educational, and environmental influences^6,7^, we predicted that renunciation of desire would account for the effects of death anxiety on mental health, but not vice versa. This study also predicted that individual differences in renunciation of desire would influence the degree to which death anxiety affects mental health. In other words, we predicted that death anxiety would mediate some of the effects of renouncing desire on mental health. We tested these predictions using a mediation model (see Fig. 1), for which it was assumed that the effect of the independent variable (renunciation of desires) on the dependent variable (mental health) is mediated by the action of the mediating variable (death anxiety). In path terms, renunciation of desires reduces death anxiety which in turn improves mental health. Confirmation of the model would support this hypothesis. Finally, we predicted that the demographic characteristics age, gender, and education would be associated with measures of death anxiety^26^ and mental health^45^, and renunciation of desires. Thus, we included age, gender and education as covariates in our analyses.

## Overview of the studies

In the present paper we report two studies. The aim of the first study was to develop the Desire Questionnaire (DQ), a measure of the renunciation of desires with five subscales: No Killing, No Harmful Speech, No Sexual Misconduct, No Intoxicants, and No Stealing. Next, we assessed the reliability and construct validity of the DQ. The aims of the second study were to provide criterion validation for the DQ by demonstrating significant relationships among renunciation of desires, death anxiety, and mental health, and to test the primary hypothesis that a significant positive effect of renunciation of desires on mental health is mediated by reduced death anxiety.

Both studies were conducted in single sessions. Two independent assistants checked data records against responses on the questionnaires to eradicate possible recording errors. Both studies were approved by the National Cheng Kung University Human Research Ethics Committee. All participants gave informed consent.

## Study 1: Construction and validation of the Desire Questionnaire

### Preliminary item selection and exploratory factor analysis

The Desire Questionnaire (DQ) was inspired by the discipline of Buddhist precepts^46^ and related publication about them^6,7,10,31,36,37,47^. We began with a pool of 29 items divided into five subscales: No Killing (8 items), No Harmful Speech (9 items), No Sexual Misconduct (3 items), No Intoxicants (3 items), and No Stealing (6 items). Responses are recorded on a Likert scale, with alternatives ranging from 1 (10% true) to 10 (100% true). The subscale scores are summed to obtain the total score. Higher scores on the DQ and each subscale indicate greater behavioural renunciation of one’s desires. All items of No Harmful Speech subscale are reverse scored.

Prior to identification of the subscales, a convenience sample of 243 volunteers (65.8% female, 32.5% atheists) ranging in age from 18 to 71 years (*M* = 35.68, *SD* = 14.75) was recruited from among students taking psychology classes at Kaohsiung Normal University and other residents of the city of Kaohsiung in Taiwan, filled out the preliminary 29-item version of the DQ (Table 1). The university sample of 94 volunteers (61.7% female, 50% atheists) ranged in age from 18 to 39 years (*M* = 21.42, *SD* = 3.02) and the community sample of 149 volunteers (68.4% female, 12.7% atheists) ranged in age from 21 to 71 years (*M* = 44.89, *SD* = 11.64), respectively. The responses were subjected to exploratory principal components factor analysis with an oblique rotation for the purpose of determining the most appropriate solution, and thus the number of factors, and then which items should be retained with this solution. An oblique rotation was used because the factors were hypothesized to be correlated. The following criteria were used to select the best model: (a) minimum eigenvalue of 1, (b) minimum factor loading of 0.30 and (c) good factor interpretability.

**Table 1:** Sample characteristics and DQ scores for the exploratory factor analysis.

	Value
**Desire Questionnaire: *M* (*SD*)**	169.42 (24.47)
Cronbach’s *α*	0.84
**No killing: *M* (*SD*)**	43.64 (11.9)
Cronbach’s* α*	0.94
**No harmful speech: *M* (*SD*)**	37.89 (10.65)
Cronbach’s *α*	0.85
**No sexual misconduct: *M* (*SD*)**	26.40 (5.24)
Cronbach’s *α*	0.84
**No intoxicants: *M* (*SD*)**	26.30 (6.7)
Cronbach’s *α*	0.80
**No stealing: *M* (*SD*)**	35.18 (5.64)
Cronbach’s *α*	0.76
*n*	243
Age range (years)	18–71 (*M* = 35.68, *SD* = 14.75)
Gender (female)	65.8%
Atheist	32.5%
**Education**	
Elementary school	0.8%
Secondary school	1.7%
High school	12.5%
University (undergraduate)	63.8%
University (postgraduate)	21.3%

Sample consisted of general adults in Taiwan.

The best model was found to be the five-factor model (see Table 2). For this model, Bartlett’s test of sphericity indicated strong correlations between the items in each subscale, $x_{190}^{2}$ = 3491.10, *p* < 0.001. The Kaiser–Meyer–Olkin value was 0.79, which is greater than 0.60, indicating that these factors are distinct and reliable^48^. Cronbach’s *α* was 0.84 for the total scale. These results justified the conduct of a confirmatory factor analysis.

**Table 2:** Factor loadings and communalities for the five subscales of the Desire Questionnaire.

Items	Factor loadings					Communality
	1	2	3	4	5	
**No killing**						
1. I would never murder someone	**0.96**	− 0.04	− 0.01	− 0.03	0.03	0.91
2. I would never commit suicide	**0.94**	0.00	− 0.01	0.02	− 0.02	0.87
3. I would never attempt to murder someone	**0.93**	0.02	− 0.02	0.00	− 0.01	0.84
4. I would never attempt to commit suicide	**0.87**	0.03	0.02	0.00	− 0.07	0.75
5. I would never attend an abortion or tell someone to abort	**0.72**	− 0.01	0.04	− 0.00	0.11	0.61
**No harmful speech**						
6. I speak in an exaggerated and rhetorical manner to convince someone	0.01	**0.82**	− 0.06	− 0.01	0.02	0.67
7. I say words to hurt someone	0.01	**0.82**	0.00	0.02	− 0.02	0.68
8. I sow discord	0.01	**0.80**	− 0.08	0.01	0.03	0.63
9. I slandered anyone	− 0.01	**0.75**	− 0.03	0.02	− 0.02	0.55
10. I speak exaggerated words to achieve my purpose most of the time	− 0.02	**0.49**	0.13	− 0.04	− 0.01	0.28
**No sexual misconduct**						
11. When I engage in a close relationship, I do not keep seeing someone else	0.03	− 0.05	**0.92**	0.01	− 0.02	0.85
12. When I engage in a close relationship, I do not have an intimate or sexual relationship with someone else	0.04	− 0.01	**0.86**	0.04	0.01	0.80
13. When I engage in a close relationship, I do not consume erotic books and films or participate in erotic chat rooms on the Internet	− 0.00	0.05	**0.61**	− 0.00	0.09	0.46
**No intoxicants**						
14. I don't smoke	− 0.02	− 0.01	− 0.07	**0.99**	− 0.01	0.94
15. I don’t use drugs	− 0.00	− 0.02	− 0.07	**0.95**	0.04	0.91
16. I don't drink	0.02	0.02	0.12	**0.46**	− 0.01	0.26
**No stealing**						
17. I am aware of the meaning of taking someone’s items or property	− 0.05	− 0.04	− 0.05	− 0.04	**0.98**	0.85
18. I can restrain myself from getting goods or positions of power in high places by intimidation and bribery	0.08	0.00	− 0.05	0.07	**0.65**	0.46
19. Before removing or taking someone’s items, I ask for his or her consent in advance	0.02	0.05	0.22	0.01	**0.49**	0.42
20. I return lost property or money to the proper owner	0.05	0.06	0.20	0.04	**0.37**	0.30
Eigenvalues	5.62	2.92	2.03	1.59	0.97	
Variance explained (%)	28.09	14.62	10.18	7.95	4.85	65.7

Table 2 presents the 20-item DQ that was used in subsequent studies. The five factors all were interpretable as expected prior to the analysis. They explain 66.7% of the item variance, with Factor 1 (No Killing) contributing 28.09% (eigenvalue = 5.88), Factor 2 (No Harmful Speech) contributing 14.62% (eigenvalue = 3.28), Factor 3 (No Sexual Misconduct) contributing 10.18% (eigenvalue = 2.29), Factor 4 (No Intoxicants) contributing 7.95% (eigenvalue = 1.79), and Factor 5 (No Stealing) contributing 4.85% (eigenvalue = 1.28). The final version of the DQ consists of 20 items, such as “I would never attempt to commit suicide” (Factor 1: No Killing) and “I don’t use drugs” (Factor 4: No Intoxicants).

With a cut off of 0.7, all five items loaded highly (> 0.72) on the first factor, four of the five items loaded highly (> 0.75) on the second factor, two of the three items loaded highly (> 0.86) on the third factor, two of the three items loaded highly (> 0.95) on the fourth factor, and one of the four items loaded highly (= 0.98) on the fifth factor.

### Confirmatory factor analysis

A confirmatory factor analysis (CFA) was performed on the five-factor model using data from a new convenience sample of 248 adults (64.8% female, 36.8% atheists) ranging in age from 18 to 84 years (*M* = 35.54, *SD* = 14.70) using AMOS 22.0 software (Table 3). They were recruited from among students taking psychology classes at Kaohsiung Normal University and other residents of the city of Kaohsiung in Taiwan. The university sample of 92 volunteers (56.5% female, 51% atheists) ranged in age from 18 to 44 years (*M* = 21.42, *SD* = 3.11) and the community sample of 156 volunteers (67.9% female, 17.9% atheists) ranged in age from 19 to 84 years (*M* = 44.14, *SD* = 12.09), respectively. Maximum likelihood was used to estimate the parameters of the model. The null hypothesis that the data would fit the model perfectly was rejected, *x*^2^_160_ = 385.25, *p* < 0.001. We used several goodness-of-fit indices to examine how accurately the present data fit the five-factor model (Table 4): The *x*^2^/df ratio was less than 3, which is considered moderate (see Wheaton, 1987); the parsimonious normed fit index (PNFI) of 0.73 also was moderate (> 0.5; see James et al. ^49^); the comparative fit index (CFI) was high (0.92); the root mean square error of approximation (RMSEA) was less than 0.08, indicating a good fit^50^; and the value for the Akaike information criterion (AIC) was substantially smaller for the independence model than for the proposed model. The fit with the five-factor model was satisfactory. Internal consistency (Cronbach’s *α*) was 0.83 for the total scale.

**Table 3:** Sample characteristics for the confirmatory factor analysis (*n* = 248).

Variable	Score
Age (years)	18–84 (*M* = 35.54, *SD* = 14.70)
Gender (female)	64.8%
Atheist	36.8%
**Education**	
University (undergraduate)	79.4%
University (postgraduate)	20.6%

**Table 4:** Goodness-of-fit indices for the five-factor model.

*x*^2^	*df*	*x*^2^/*df*	PNFI	CFI	RMSEA	RMSEA 90% CI	AIC	
							Five-factor model	Independent
358.25**	160	2.40	0.73	0.92	0.07	[0.06, 0.08]	485.25	3043.6

*AIC* Akaike information criterion, *CFI* comparative fit index, *PNFI* parsimonious normed fit index, *RMSEA* root mean square error of approximation. **p < 0.01.

The DQ was validated by both the exploratory factor analysis and the confirmatory factor analysis. The items were the same for each analysis, but the samples were different. As the analyses provided evidence for adequate psychometric validity of the DQ, we used it in Study 2.

### Study 2

The aim of the second study was to test for possible mediation by death anxiety of a possible relationship between renunciation of desires and mental health. There were four specific hypotheses:

- Hypothesis 1: Renunciation of desires is negatively associated with death anxiety.
- Hypothesis 2: Renunciation of desires is positively associated with mental health.
- Hypothesis 3: Death anxiety is negatively associated with mental health.
- Hypothesis 4: (Low) death anxiety partially mediates a positive effect of renouncing desires on mental health.

## Method

### Participants

A convenience sample of 501 volunteers was recruited from among students taking psychology classes at Kaohsiung Normal University and other residents of the city of Kaohsiung in Taiwan. Six were removed from the sample for missing data, leaving a final sample of 501 (66.1% female, 27.8% atheists) ranging in age from 18 to 85 years (*M* = 35.58, *SD* = 14.76) (Table 5). The university sample of 194 volunteers (59.2% female, 47.4% atheists) ranged in age from 17 to 44 years (*M* = 21.53, *SD* = 3.21) and the community sample of 307 volunteers (71.3% female, 15.3% atheists) ranged in age from 19 to 85 years (*M* = 44.72, *SD* = 11.88), respectively.

**Table 5:** Descriptive data: Sample characteristics, means and standard deviations of psychological measures (*N* = 501).

Variable	Score
Age (years)	17–85 years (*M* = 35.58, *SD* = 14.76)
Gender	66.1% female
Atheists	27.8%
**Education**	
Undergraduate	60.9%
Postgraduate	21.5%
Desire Questionnaire	169.89 (23.20), Range 62.92–200
Death Anxiety Scale	41.88 (8.85), Range 17–69
Chinese Health Questionnaire	2.87 (1.90), Range 0–11

For continuous variables, the mean is followed by the standard deviation in parentheses.

### Measures

The demographic variables were age, gender, and education, and religion. The education levels were elementary school, junior high school, high school, college, graduate school, and postgraduate. The religions were Christianity, Buddhism, traditional Chinese religions, and atheism.

### Desire Questionnaire

We used the Desire Questionnaire developed in Study 1.

### Death Anxiety Scale

We used the Death Anxiety Scale (DAS), developed by Templer^51^ and widely employed to measure death anxiety^52^. We used the Chinese translation of the DAS, which has good reliability and validity^53^. The DAS has 15 items rated as “true” (1) or “false” (0), with a higher score indicating greater death anxiety. However, some researchers suggested to use a five-point Likert scale with response alternatives of least anxiety (1) and most (5) to reflect real situation^54^. For this reason, we adopted the five-point Likert scale. We performed an extra analysis to demonstrate a satisfactory internal consistency (Cronbach’s α) of 0.76 for the DAS.

### Chinese Health Questionnaire (CHQ)

As our measure of mental health, we used the 12-item Chinese Health Questionnaire (CHQ), which was developed as a screening measure for minor psychiatric illness^55^. The CHQ is a modification based on the concepts and structure of the General Health Questionnaire (GHQ)^56^ and reflecting cultural differences between Chinese and Western culture^57^. Participants respond to the 12 items on a 4-point Likert scale with response alternatives of “not at all” and “same as usual” (both scored as 0), and “more than usual” and “a lot more than usual” (both scored as 1), with a higher score indicating more severe psychiatric symptoms. The CHQ demonstrated an internal consistency (Cronbach’s α) of 0.79^55^.

### Procedure

A research assistant informed participants of the nature of the research. Participants received a booklet including the demographic items, followed (in order) by the DQ, CHQ, and DAS.

### Data analysis

Means, standard deviations, and percentages were calculated for the demographic variables. Pearson correlation coefficients were calculated to measure the pairwise linear relationships between scores on the DS, DAS and CHQ. To test the mediation, we used stepwise multiple regression and Sobel test.

### Ethical approval

The two studies and their relevant details were approved by the Human Research Ethics Committee of National Cheng Kung University in Taiwan. All studies were performed in accordance with relevant guidelines and regulations. The volunteer participants were informed of the nature of the research and given assurances of confidentiality; the researchers then obtained their informed consent.

### Informed consent

Informed consent was obtained from all individual participants included in the study.

## Results

### Descriptive data

Descriptive data for Study 2 are presented in Table 5.

### Correlational analyses

The partial correlations between the Desire Questionnaire, The Death Anxiety Scale, and the Chines Health Questionnaire, controlling for age, gender, and education, are shown in Table 6. As expected, the DQ had a significant negative correlation with the DAS and a significant negative correlation with the CHQ (a higher score indicating more severe psychiatric symptoms). The DAS had a significant positive correlation with the CHQ. These results support Hypotheses 1, 2, and 3.

**Table 6:** Partial correlations between Desire Questionnaire, Death Anxiety Scale, and Chinese Health Questionnaire scores, controlling for age, gender, and education.

	Death Anxiety Scale	Chinese Health Questionnaire
Desire Questionnaire	− 0.11*	− 0.22***
Death Anxiety Scale		0.14**

*p < 0.05, **p < 0.01, ***p < 0.001 (two-tailed).

### Mediation analyses

We conducted a linear mediation analysis to determine whether death anxiety acted as a partial mediator of the effect of renouncing desires on mental health. Figure 1 presents a schematic of the model and Table 7 summarizes the results of each step. Step 1 estimated the “c” path by regressing CHQ scores on DQ scores. Step 2 estimated the “a” path by regressing DAS scores on DQ scores. Step 3 estimated the “b” path by regressing CHQ scores on DAS scores controlling for DQ scores. Step 4 estimated the “c” path, which distinguishes between partial and complete mediation based on whether the demonstrated effects of renunciation of desire on mental health are reduced to zero when DAS scores are included as a predictor in the equation.

**Table 7:** Summary of analysis of the effect of renunciation of desire (DQ scores) on mental health (CHQ scores) mediated by death anxiety (DAS scores).

Predictor	Mediator	Step 1 Path c	Step 2 Path a	Step 3 Path b	Step 4 Path c’	Sobel test
DQ (total)	DAS	− 0.19***	− 0.09*	0.13***	− 0.18**	− 1.82*

The values in the table are standardized regression coefficients (*β*). ***p < 0.001, **p < 0.01, *p < 0.05.

As seen in Table 7, the relationship between DQ and CHQ scores is partially mediated by DAS. These results support our Hypothesis 4.

## General discussion

The two studies presented in this article were designed to develop the DQ as a measure of renunciation of desire and use it to test the relationships of renunciation of desire with death anxiety (measured by DAS) and mental health (measured by CHQ). Study 1 provided evidence for the psychometric adequacy and validity of the DQ by exploratory factor analysis and confirmatory factor analysis. The DQ was shown to be a reliable and valid instrument for use with the general adult population in Taiwan. In Study 2, the significant negative correlation of DQ with DAS and the significant positive correlation of DQ with CHQ demonstrated the criterion validity of DQ as measuring a construct (renunciation of desire) that is relevant to mental health. The results of these analyses demonstrate satisfactory construct and criterion validity for the DQ.

The second study was the first to investigate the relationships among renunciation of desires, death anxiety, and mental health. As expected, renunciation of desires had a positive association with mental health and a negative association with death anxiety. Our finding of a significant negative correlation of death anxiety with mental health is consistent with previous studies^40–42^.

Death anxiety was found to partially mediate the relation between renunciation of desires and mental health. Consistent with our prediction, these findings suggest that the effect of renouncing desires on mental health can be accounted for at least partially by the effects of reduced death anxiety caused by renouncing desires. Though the role of death anxiety in mediating the relationship between renunciation of desires and mental health was first discovered in the present study, this finding needs to be replicated before definitive conclusions can be drawn.

The present study supports the Nonself Theory^6^ as a theory of death anxiety and shows its relevance to nonself and mental health that Buddhism provides an alternative perspective on the meaning of death and the Buddhist tenet that self is the main cause of death anxiety for more than 2500 years^7,31^. Undoubtedly, everyone experiences death anxiety at some time in life, but it can be reduced or overcome by moving self towards the nonself state by severing all ties to the world, renouncing unnecessary personal desires^30^, and practicing the Buddhist wisdom. According to Buddhism, death serves as both a continuous reminder that life is finite; recognition of this directly leads to the search for the meaning of life, conducted mainly by cultivating self and moving it towards the nonself state as a way to overcome the death anxiety. Our results demonstrate that although people indeed perceive death as a threat, it is not necessary as the time of death approaches to invoke a defensive response by moving mortality away from our focal awareness or appealing to our self-esteem, as described by TMT^26–28^. But avoiding this requires that one adopt a different explanation for the meaning of death. Our research can help in this regard by providing evidence for an alternative framework, based on Buddhism, for understanding at least some of the effects of the salience of mortality.

### Limitations and suggestions for future research

The DQ is still in its infancy and our results suggest a number of opportunities for further investigation. The subjects for its validation were recruited from Chinese societies, and we suggest that further research on the DQ be conducted with more diverse samples so as to generalize the findings cross-culturally. Moreover, our results can be generalized only to healthy individuals, and future studies on specific disease groups would be valuable. Although we found significant interrelationships among renunciation of desires, death anxiety, and mental health, questions remain about how these three factors are related with each other in different cultures. In particular, the concept of the renunciation of desires deserves further investigation in different cultures because it can also be found across different cultures^58^, and attention should be focus on deepening our understanding of how Buddhist wisdom enhances mental health, and especially whether this wisdom is applicable exclusively in Buddhist cultures. Based on the Nonself Theory^6^, the wisdom of Buddhism provides a sophisticated framework for explaining a possible mechanism for this phenomenon of renunciation of desires, as well as its benefits for mental health, which is an important topic for further empirical investigation. Even more so, investigation of how development of the avoidance of desire-driven pleasures promotes the nonself should be a next step in this line of research. Future research could also profitably pay more attention to clinical application of this avoidance, for example, on whether the avoidance of desire-driven pleasure is related to the successful application of strategies for coping with events that lead to an increase in death anxiety. Some might think our results support TMT. For example, based on the TMT, by following the five precepts, one might feel that he/she is living up to the standards of value inherent in his/her cultural worldview and therefore resulted in higher self-esteem. However, based on the Buddhism, the purpose of obeying the five precepts is not to boost self-esteem. More studies are needed to approve or disapprove this issue. Furthermore, future research should consider the effect of social desirability response. For example, one might increase their prosocial response while filling out the questionnaire. Finally, precisely how reduction of desires promotes good mental health is still not clear, making this another important topic for future studies. The correlations between DQ, death anxiety, and mental health in Study 2 are small in magnitude. Although statistically significant, they still might be due to chance. More studies are needed to rule out this possibility.

## Conclusion

The purpose of the two studies reported in this paper was to demonstrate the role of renunciation of desire in the reduction of death anxiety and promotion of mental health. As mentioned earlier, based on Nonself Theory^6^, a reduction in the level of desire linked to the nonself state leads to reduced death anxiety and improved mental health. These results also support the Nonself Theory as a theory of death anxiety and show its relevance to the relationship between nonself and mental health. Although there have been many studies of Buddhism, which has been widely practiced for more than 2500 years, the majority of them have been focused on meditation and its effects, such as increased emotional stability, heightened positive emotion, and improved attention. There have been few empirical studies or theories directly relating Buddhist teachings to lifestyle issues. The present research is among the first attempts to study Buddhist teachings in relation to death anxiety in this difficult time of pandemic. Our hope is that this research has helped to fill these gaps, because it suggests that Buddhism provides a reliable and useful way to cope with life’s adversities, which contribute to a variety of mental health problems. Further research on these attributes could open significant new avenues for mental health research and unravel the secrets of why Buddhism has lasted for thousands of years.

## Data Availability

The datasets analyzed during the current study are available from the corresponding author on reasonable request.
